# Sensitivity Enhancement of Polymer Optical Fiber Surface Plasmon Resonance Sensor Utilizing ITO Overlayer

**DOI:** 10.3390/s25061863

**Published:** 2025-03-17

**Authors:** Getinet Woyessa, Ole Bang

**Affiliations:** DTU Electro, Department of Electrical and Photonics Engineering, Technical University of Denmark (DTU), DK-2800 Kongens Lyngby, Denmark; oban@dtu.dk

**Keywords:** surface plasma resonance, polymer optical fiber, gold, indium tin oxide, refractive index sensor, biosensor

## Abstract

We present an experimental study of a sensitivity-enhanced surface plasmon resonance (SPR) sensor utilizing a cladding etched multimode polymer optical fiber (POF) coated with a layer of gold followed by an indium tin oxide (ITO) layer. Our findings indicate that POF SPR sensors with an ITO overlayer exhibit higher sensitivity compared to those coated solely with gold. Additionally, increasing the thickness of the ITO layer increases the sensitivity of the sensor at the expense of a broader SPR spectrum. We determined that the optimal ITO thickness for maximizing sensitivity is 25 nm. The sensor coated with 40 nm gold and 25 nm ITO demonstrated a refractive index sensitivity of 2258 nm per refractive index unit (nm/RIU) with a figure of merit and resolution of 10.13 ${RIU}^{-1}$ and $2.74\times 10^{-4}\;RIU$, respectively, within the range of 1.33 to 1.37 RIU. Notably, this sensitivity is 70% greater than that of a POF SPR sensor coated only with 40 nm gold. Long-term stability tests conducted in a hydrated environment confirmed that the ITO layer remains unaffected over time and that the maximum SPR wavelength drift was only 1.2 nm. The standard deviation of the three-round measurements also revealed that the sensor has good repeatability. We believe that this sensor offers a simple structure and a relatively easy fabrication process, eliminating the need for side polishing while providing a large interaction area, making it a promising candidate for high-sensitivity biosensing applications.

## 1. Introduction

Surface plasmonic resonance (SPR)-based sensors are suited for the detection of analytes relevant to clinical diagnostics, environmental sciences, infectious diseases, food, and industrial applications due to their high sensitivity, label-free and real-time responses, and simple procedures [1,2]. The sensing scheme of SPR sensors was initially based on optical prisms, where surface plasmons were excited at the interface of a metallic film coated on the base of a prism and the surrounding dielectric medium by attenuated total reflection [3]. While prism-based SPR sensors are effective for a variety of applications, their limitations in size, complexity, and portability restrict their effectiveness in field applications and remote sensing. To address these limitations, optical fibers have been introduced as a replacement for prisms [4]. Fiber optical SPR sensors offer a simplified design, enhanced miniaturization, portability, and a high degree of integration, making them suitable for real-time and in situ monitoring compared to bulk optical systems. Additionally, while prism-based SPR sensor systems typically use angular interrogation methods, fiber optical SPR sensors employ spectral interrogation techniques [5,6]. These advancements have facilitated widespread applications, including the screening of viral and bacterial infections, the detection of specific toxins in food processing, and the monitoring of water quality in urban supply systems [7]. The integration of SPR technology with optical fibers has been extensively utilized for sensing a wide range of parameters, such as fluid refractive index (RI), film thickness, surface roughness, pH, temperature, urea, glucose, and different pollutants in water and air [8].

The majority of fiber optical SPR sensors developed to date are based on silica optical fibers, primarily due to their maturity and wide availability. However, in the past 10 years, significant progress has been made in the development of polymer optical fiber (POF)-based SPR sensors. POFs offer several unique advantages over silica fibers, including ease of handling, high flexibility, superior coupling efficiency, and biocompatibility. Additionally, POFs can be easily processed and modified, even in larger diameters, which is advantageous for fabricating SPR sensor probes. Moreover, gold (Au) films exhibit excellent adhesion to polymer surfaces, eliminating the need for an intermediate chrome layer, which is often required with silica fibers. As a result, numerous POF-based SPR sensors have been reported in the literature [9,10,11,12,13,14,15]. Despite this progress, there have been limited efforts made to enhance the sensing performance, particularly the sensitivity of the POF SPR sensors. Current sensitivity enhancement techniques include depositing a thin high-index polymer layer between the core of a side-polished POF and the Au film [9]; tapering the fiber followed by side polishing the waist region [16]; bending the fiber into a U-shape and side polishing the bend region [17]; and double-sided polishing [18]. However, these methods often involve tedious polishing processes that can be less repeatable unless high-precision polishing equipment is used. Additionally, tapering and bending the fiber can compromise the sensor’s compactness and robustness.

In this article, we present a sensitivity-enhanced POF-based SPR sensor, achieved by etching the cladding of a multimode POF and coating it with a Au film, followed by an indium tin oxide (ITO) overlayer. We experimentally compare the RI sensing performance of POF SPR sensors coated solely with Au against those with the additional ITO layer of varying thickness. Additionally, we evaluate the adhesion of the ITO film to the Au surface and its stability under prolonged exposure to a hydrated environment.

## 2. Materials and Methods

The POF used to fabricate the sensor is a step-index multimode ESKA POF from Edmund Optics. The core and cladding diameters of the fiber are 486 µm and 500 µm, respectively, resulting in a cladding thickness of only 7 µm. The core material is acrylic polymer PMMA (polymethyl-methacrylate), while the cladding is fluorinated polymer, with respective RIs of 1.492 and 1.402.

**Figure 1:**
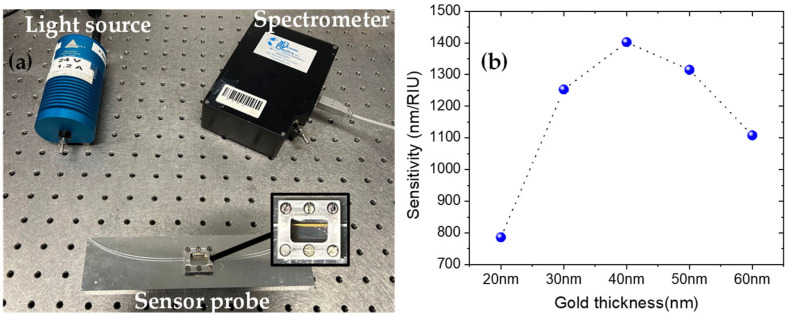
(**a**) Characterization setup for the etched POF-based SPR sensor. (**b**) The sensitivity of the sensor solely with gold layer, versus the thickness of the gold layer.

To fabricate the sensor, a 50 cm long fiber is mounted in a holder made of aluminum, with an opening length of 10 mm, as shown in Figure 1a. Then, by dropping Dimethyl Sulfoxide into the opening of the holder, the cladding is etched away over the exposed 10 mm long section of the fiber. The cladding is completely etched away in less than 5 min. Dimethyl Sulfoxide does not dissolve acrylic, so the core remains unaffected during the etching process. The etched section enables access to the evanescent waves when light passes through the core of the fiber, which is essential for exciting the surface plasmon. In this way, six fiber samples were prepared. Next, the etched section of each fiber is coated with Au using a sputter coater (Q150T Plus, Quorum, Sussex, UK). The sputter coater is fitted with a quartz crystal thickness monitor, which allows us to measure the thickness of the film in real time during the coating process. The desired thickness of the film is set, and the built-in quartz crystal monitor measures the thickness, stopping the process once the required thickness is reached. This ensures precise control over the film thickness. The thickness of the Au coated on each sample was 40 nm, which was chosen because our previous experiments showed it provided the highest sensitivity, as shown in Figure 1b. Following Au coating, five of the fiber samples were coated with 10 nm, 15 nm, 20 nm, 25 nm and 30 nm of ITO. One of the samples is not coated with ITO for comparison. It is worth mentioning that, to guarantee the uniformity of the Au and ITO films throughout the deposition process, the fibers were rotated at a speed of 70 rad/s, maintaining a turbo sputter bleed vacuum of $5\times 10^{-3}$ mbar and a sputter current of 20 and 100 mA, respectively. In addition, both targets were obtained from Quorum.

To characterize the sensors, both ends of the fiber samples were connected with SMA connectors and polished, which allowed us to easily connect the fibers to the light source and the spectrometer. The light source is a halogen lamp (HL-2000, Ocean Optics, FL, USA) with a wavelength emission range of 360 nm to 1700 nm and an output power of 63 mW. The spectrometer (HR2000+, Ocean Optics) has a detection range of 200–1100 nm with a resolution of 0.62 nm. The spectrometer is connected to a computer for data acquisition. Figure 1a shows the experimental setup used to characterize the sensor. To measure the RI sensitivity of the sensors, glucose solutions of different RIs ranging from 1.33 to 1.37 with a step of 0.01 were prepared. An Abbe refractometer with a resolution of 0.0005 is used to measure the RIs while preparing the solutions. The test is performed by dropping the solutions from the top of the fiber holder opening. Before a new solution is tested, the previous solution is rinsed with distilled water until the original air spectrum is recovered. It is worth mentioning that all the experiments in this work were performed at ambient temperature and relative humidity. The spectra are measured in the following sequence: first, a reference spectrum is measured in air. Then, the background spectrum is measured when the light source is off. Finally, the sample spectra are measured for different RI glucose solutions. The SPR transmission spectrum is obtained by dividing the difference in the sample and the background spectrum by the difference in the reference and the background spectrum. This gives us the SPR spectrum, with the characteristic being a dip in transmission, indicating a loss of energy due to the transfer of energy from the light to the surface plasmons.

## 3. Results

**Figure 2:**
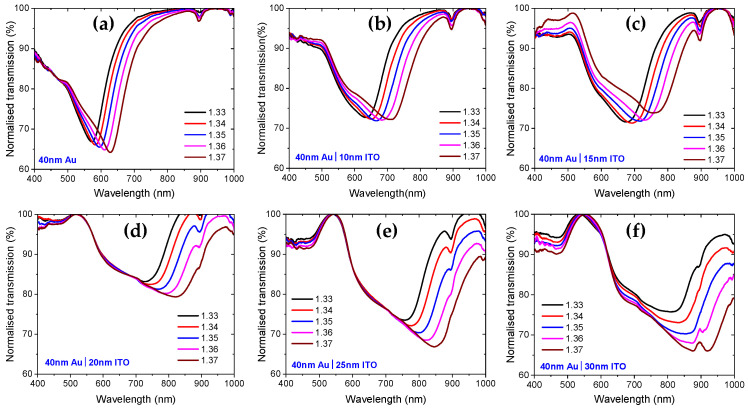
Normalized transmission versus wavelength for RIs ranging from 1.33 to 1.37 for (**a**) only 40 nm Au, (**b**) 40 nm Au and 10 nm ITO, (**c**) 40 nm and Au 15 nm ITO, (**d**) 40 nm Au and 20 nm ITO, (**e**) 40 nm Au and 25 nm ITO, and (**f**) 40 nm Au and 30 nm ITO-coated etched POF-based SPR sensor.

**Figure 3:**
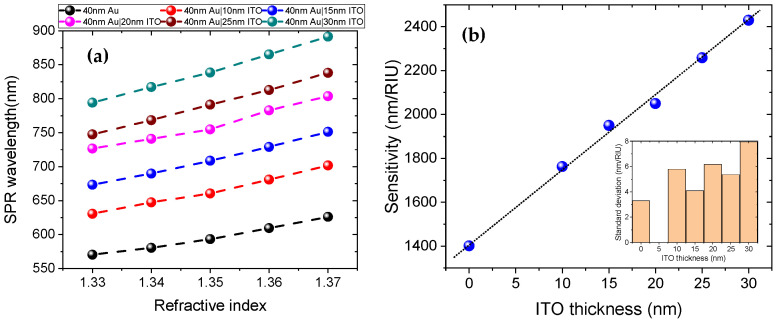
(**a**) SPR wavelength versus RI for different ITO film thicknesses. (**b**) The sensitivity versus ITO film thickness. Inset: The standard deviation of the sensitivities for each ITO thickness. Data are reported as the mean value of three measurements.

Figure 2 shows the SPR transmission spectra, and Figure 3a presents the SPR wavelength versus RI for RIs ranging from 1.33 to 1.37. The sensitivity of each sensor is reported in Figure 3b, which is a linear fit of the data in Figure 3a, with R-squared greater than 0.99 for each sensor. All sensors coated with an ITO overlayer exhibited higher RI sensitivity compared to the sensor coated only with Au. Additionally, as the thickness of the ITO layer increases, the sensitivity also increases, as demonstrated in Figure 3b.

**Figure 4:**
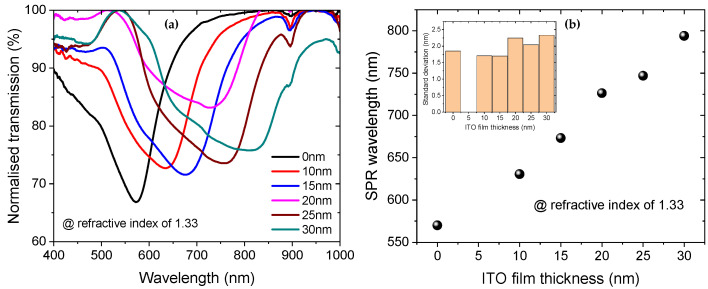
(**a**) Normalized transmission versus wavelength for different ITO film thicknesses for a fixed RI of 1.33, showing a red-shift in the resonance wavelength with increasing ITO layer thickness. (**b**) The tracking of the SPR wavelength versus ITO film thickness for a RI of 1.33. Inset: The standard deviation of the SPR wavelength for each ITO thickness for a RI of 1.33. Data are reported in (**b**) as the mean value of three measurements.

Importantly, as can be seen from Figure 2, the SPR transmission dips broaden with increasing ITO layer thickness, which can reduce the sensor’s detection accuracy. Furthermore, SPR wavelength is red-shifting into the high loss region of the POF as the thickness of the ITO layer increases, as seen from the general spectra in Figure 2 and the result for a fixed RI of 1.33 in Figure 4. As a result, we evaluate that the ITO film thickness for sensitivity enhancement should not exceed 25 nm. For this sensor design, the sensitivity enhancement is 70% higher than that of the sensor coated with only 40 nm Au. Within the range of 1.33 to 1.37 RIU, the refractive index sensitivity of the sensor with a 25 nm ITO overlayer is 2258 nm/RIU, with a full width at half maximum (average for all RIs), a figure of merit, and a resolution of 219 nm, 10.13 ${RIU}^{-1}$ and $2.74\times 10^{-4}\;RIU$, respectively. These performance parameters are calculated by the formulas defined in reference [19]. It is also important to note that as the thickness of the ITO layer increases, the resonance wavelength shifts to longer wavelengths, as shown in Figure 4. For a RI of 1.33, the SPR for the sample coated with only 40 nm Au is 570.11 nm, while for the sample coated with 40 nm Au and 25 nm ITO, it is 746.79 nm, resulting in a tuning of about 176.68 nm.

**Figure 5:**
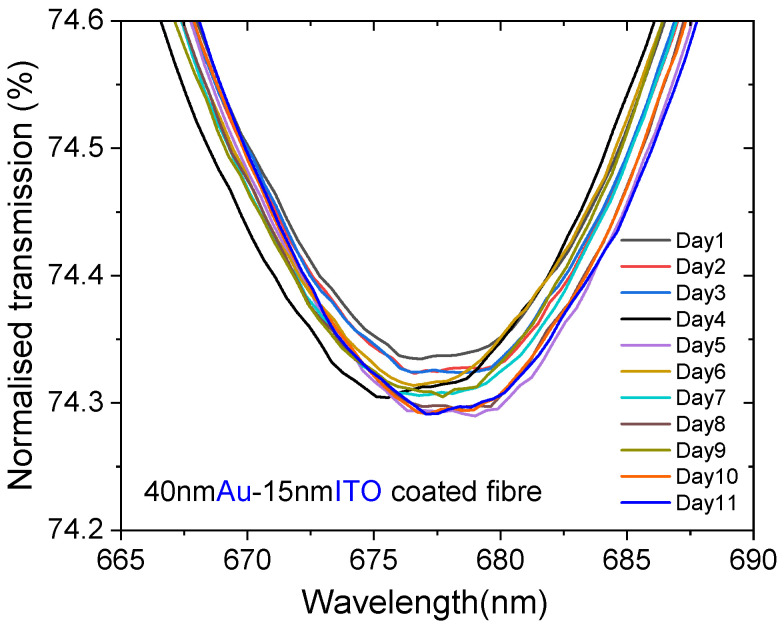
Stability measurement over 11 days performed with the sensor coated with 40 nm Au and 15 nm ITO fully immersed in distilled water.

In addition, we have investigated the adhesion of the ITO film to the Au surface and its stability under prolonged exposure to a hydrated environment. This investigation was performed using a sensor coated with 40 nm Au and 15 nm ITO. The sensor was exposed to distilled water for 6 h each day over 11 consecutive days, and the SPR spectrum was recorded at the end of the 6 h period each day, as shown in Figure 5. The maximum resonance wavelength deviation was observed for the spectra recorded on days 4 and 11, which is only 1.2 nm, indicating good stability of the dual-layer sensor. This is due to the fact that ITO exhibits excellent stability similar to gold under atmospheric conditions and an aqueous environment [20]. To our knowledge, this is the first report on the long-term stability measurement of POF-based SPR sensors over several days.

## 4. Discussion

The sensitivity of an SPR sensor is influenced by the intensity of the electric field at the surface and the interaction volume with the analyte [21]. In the sensor wavelength operation region 400–900 nm, the real part of the RI of ITO is approximately 1.813, which is larger than the RI of the core of the POF (1.492) and the gold film (<1). This high-index layer enhances the electric field intensity at the interface between the ITO layer and the surrounding medium (the sample), thereby facilitating stronger interactions between the evanescent field and the analyte compared to that without the ITO layer, thereby increasing sensitivity [22]. This mechanism of sensitivity enhancement is also observed in silica fiber-based SPR sensors coated with a silver and silicon overlay (80% enhancement) [23], as well as with copper and oxide overlayers (a factor of 2.5 enhancement) [24]. As shown in Figure 4, as the thickness of the ITO layer increases, the SPR wavelength shifts to longer wavelengths. This is attributed to (1) the RI of ITO increases as the thickness increases on a nanometer scale [25]; (2) the overall effective RI of the region above the Au layer increase as the volume fraction of the ITO increases, i.e., as its thickness increases within the decay length of the evanescent field [26]. These changes in the effective RI affect the phase-matching conditions for the surface plasmons. Specifically, a thicker ITO layer results in a higher effective refractive index, shifting the resonance condition to longer wavelengths. This phenomenon of wavelength tuning is also observed with silica fiber-based SPR sensors coated with silver and silicon overlay as the thickness of the silicon increases [23]. The sensitivity enhancement with increasing ITO thickness is also related to the increase in RI of the ITO and the wavelength tuning to a longer wavelength. As the RI of the ITO increases the electric field intensity is enhanced, and as the resonance wavelength increases, the penetration depth of the SPR sensor also increases, thereby enhancing the interaction volume with the analyte and improving the sensitivity [27,28,29]. It is important to note that increasing the thickness of the ITO layer can introduce additional optical losses due to scattering and absorption, resulting in a broader resonance peak [8,23,26]. This broadening effect is similar to what has been observed in the literature when increasing the length of the SPR probe [19]. In our experiments, we also specifically observed this broadening phenomenon as the ITO thickness was increased, as shown in Figure 4a. It is also worth mentioning that the wavelength tuning feature is also advantageous for developing dual- or multi-parameter SPR sensors [30,31,32]. The sensitivity enhancement achieved in this work is higher than that of other sensitivity enhancement mechanisms reported in the studies such as side polished POF with buffer layer (5% enhancement) [9], and tapered side polished POF (28% enhancement) [16]. We would like to emphasize that the diameter of the fiber used in our work is 500 µm, whereas in [9,16], it is 1000 µm. A study by cennamo et al. reported that the diameter of the fiber affects the sensor performance parameters [33]. Their results indicated that increasing the fiber core diameter enhances sensitivity and resolution, and decreases the signal-to-noise ratio. Additionally, our sensor design is straightforward and easy to fabricate, as it does not require tapering or polishing the fiber. It is also robust, since only 7 µm cladding layer is removed from a 500 µm diameter fiber.

## 5. Conclusions

In this article, we present a sensitivity-enhanced POF-based SPR sensor, achieved by etching away a thin cladding layer of a multimode POF and subsequently coating it with a layer of Au, followed by ITO. We experimentally compared the RI sensing performance of sensors coated solely with Au against those with varying ITO thicknesses. The results revealed a 70% increase in sensitivity for the sensor with 40 nm Au and 25 nm ITO, compared to the sensor with only a 40 nm layer of Au (optimized thickness with maximum single Au layer sensitivity), within the RI range of 1.33 to 1.37. This sensor design exhibited a refractive index sensitivity, full width at half maximum (average for all RIs), a figure of merit and a resolution of 2258 nm/RIU, 219 nm, 10.13 ${RIU}^{-1}$ and $2.74\times 10^{-4}\;RIU$, respectively, within the range of 1.33 to 1.37 RIU. Additionally, we evaluated the adhesion of the ITO film to the Au surface and its stability by exposing it to distilled water for 6 h each day over 11 consecutive days. This showed a maximum resonance wavelength deviation of only 1.2 nm, indicating good stability of the dual-layer sensor. The standard deviation of the three round measurements also revealed that the sensor has good repeatability, with a deviation of 6 nm/RIU for the sensitivity and 2 nm for SPR wavelength shift. Moreover, our sensor is easy to fabricate, as it eliminates the need for side polishing or tapering of the fiber. Notably, the cladding etching removes only 1% of the fiber, ensuring mechanical stability. Furthermore, because the fiber is etched and coated on all sides, unlike side-polished SPR sensors, it offers a larger active area for the functionalization of biomolecules in bio-sensing application without increasing the sensor’s length.

## Data Availability

Data underlying the results presented in this paper are not publicly available at this time but may be obtained from the authors upon reasonable request.
